# Olfactory impairment in posterior cortical atrophy

**DOI:** 10.1136/jnnp-2012-304497

**Published:** 2013-02-23

**Authors:** Pirada Witoonpanich, David M Cash, Timothy J Shakespeare, Keir X X Yong, Jennifer M Nicholas, Rohani Omar, Sebastian J Crutch, Martin N Rossor, Jason D Warren

**Affiliations:** 1 Dementia Research Centre, UCL Institute of Neurology, University College London, London, UK; 2 Centre for Medical Image Computing, University College London, London, UK; 3 Department of Medical Statistics, London School of Hygiene and Tropical Medicine, London, UK

**Keywords:** Smell, Psychology, Experimental, Dementia, Alzheimer's Disease, MRI

## Introduction

Olfactory dysfunction develops in many neurodegenerative diseases, and is an early feature of the most common neurodegenerative disorder, Alzheimer's disease (AD).^1–5^ Anatomically, the central olfactory pathways traverse brain regions implicated in the common neurodegenerative diseases, including the mesial temporal and inferior frontal lobes.^6–10^ Phenotypically, AD shows substantial diversity with several important variant syndromes, notably posterior cortical atrophy (PCA),^11^ which is underpinned by AD pathology in over 70% of cases across series. Olfactory impairment in PCA might act as an early signal of underlying AD pathology in these clinically atypical cases; while if olfactory processing were spared in PCA, this would imply that olfaction depends chiefly on disease topography. However, there is presently very little information concerning olfaction in PCA.

Here we compared olfactory function prospectively in cohorts of patents with PCA and typical AD (tAD). Neuroanatomical associations of odour identification were assessed using voxel-based morphometry (VBM). We hypothesised that PCA would be associated with olfactory impairment qualitatively similar to tAD, but less severe (reflecting differential involvement of olfactory cortex); and that deficits of odour identification in both syndromes correlate with grey matter loss in anteromedial temporal and inferior frontal lobes.^2^ ^6–10^

## Methods

Fifteen patients fulfilling consensus criteria for PCA,^11^ 10 patients fulfilling The National Institute of Neurological and Communicative Disorders and Stroke and the Alzheimer's Disease and Related Disorders Association (NINCDS-ADRDA) criteria for tAD and 32 healthy control (HC) subjects participated. Cerebrospinal fluid (CSF) measurements, available for four patients with PCA, revealed a raised total-τ:β-amyloid ratio (>1) in each case, consistent with underlying AD. Informed consent was obtained from all subjects and the study had local ethics committee approval.

All subjects had a comprehensive general neuropsychological assessment which corroborated the clinical impression in both disease groups (see online supplementary table S1). Further details about the behavioural assessments are in online supplementary material.

Olfactory processing was assessed using the 40-item, four-alternative-forced-choice University of Pennsylvania Smell Identification Test (UPSIT: British version).^12^ We modified the standard UPSIT in two ways: on each trial, the subject was asked to categorise the source of the odour as edible or inedible (see online supplementary table S2) before identifying it; and target-foil choices were name-picture combinations rather than odour names alone, to maximise available response cues. Group differences were assessed using analysis of variance (ANOVA) or χ^2^ tests (Stata V.12.1), adjusting for cognitive severity, verbal processing measures, age and gender.

Twelve patients with PCA and eight patients with tAD had T1-weighted volumetric magnetic resonance (MR) brain images acquired on a 3.0T Siemens Trio scanner. VBM was performed using SPM8 (http://www.fil.ion.ucl.ac.uk/spm) following previously described procedures (see online supplementary material). Linear regression was used to examine voxel-wise associations between regional grey matter volume and odour identification performance (age-normed and gender-normed percentile score) across the combined patient cohort, within the PCA subgroup, and between syndromic subgroups, incorporating syndromic group, mini-mental state examination (MMSE) score and total intracranial volume as covariates. Statistical parametric maps were assessed thresholded over the whole brain volume and after multiple-comparisons correction over small volumes of interest (right and left anteromedial temporal lobes and orbitofrontal cortex) specified in our prior anatomical hypotheses.

## Results

**Figure 1:**
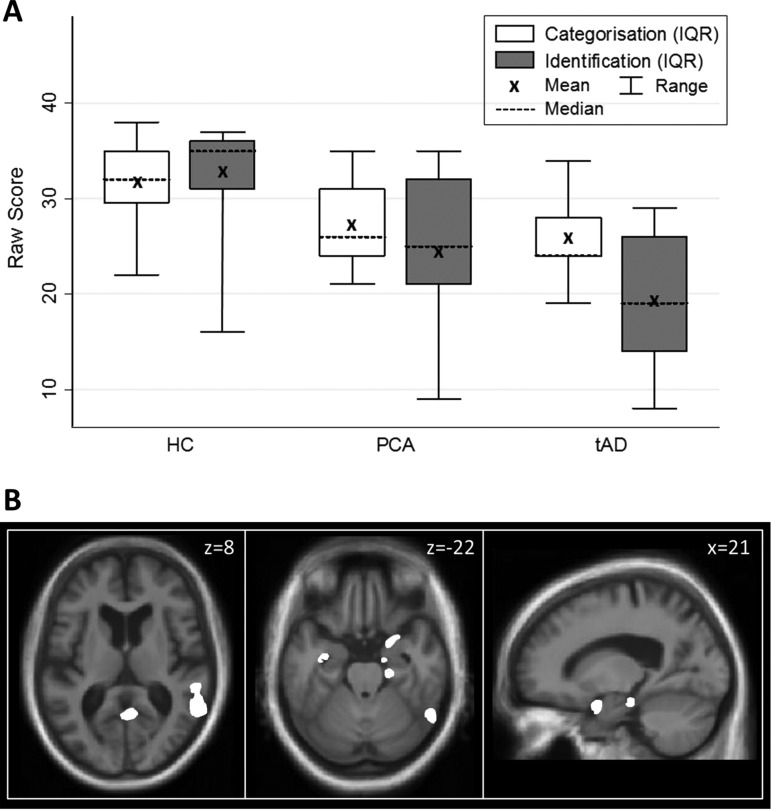
Summary of behavioural and neuroanatomical findings. (A) Distribution plots of olfactory performance comparing mean, median, IQR and full range of odour categorisation and identification of raw scores of subjects in the healthy control (HC), posterior cortical atrophy (PCA) and typical Alzheimer's disease (tAD) groups. (B) Statistical parametric maps (SPMs) of regional grey matter atrophy associated with odour identification performance across the combined PCA and tAD cohorts. SPMs are shown rendered on axial (left, middle panels) and sagittal (right panel) sections of the mean normalised structural T1-weighted brain MR image. The axial sections show the right hemisphere on the right; the sagittal section is through the right hemisphere. For display purposes, SPMs have been thresholded at p<0.001 uncorrected over the whole brain volume; see online supplementary table S3 for associations attaining significance after multiple-comparisons correction. The plane of each section is shown in Montreal Neurological Institute (MNI) coordinates in millimetres (mm).

For both patient groups, mean odour categorisation and identification raw scores were significantly lower than the HC group (p<0.001; figure 1A, online supplementary table S1; individual data in online supplementary figure S1). Based on published UPSIT norms,^12^ four patients with PCA (26%) and three patients with tAD (30%) scored <5th percentile. Mean raw or percentile scores did not differ significantly (p>0.1) between the PCA and tAD groups. After correction for guessing, mean odour identification scores were higher than mean categorisation scores for PCA patients as well as HC subjects (see online supplementary table S1). An error analysis of individual odour items in the identification test revealed a qualitatively similar profile of errors across all groups (see online supplementary figure S2).

Statistical parametric maps of significant regional grey matter associations of odour identification performance are displayed in figure 1B (quantitative data summarised in online supplementary table S3). Across the combined patient cohort, performance on the odour identification task was positively associated with regional grey matter volume in right entorhinal cortex and parahippocampal gyrus (p<0.05 FWE-corrected over the temporal lobe volume of interest). At a more lenient threshold (p<0.001 uncorrected over the whole brain volume), additional associations were present in more distributed, predominantly right-sided cerebral areas, including hippocampus, posterior inferior temporal gyrus/sulcus, temporo-parieto-occipital junction and premotor cortex (see online supplementary table S3). Similar grey matter associations of odour identification performance were identified for the PCA subgroup alone (p<0.001 uncorrected over the whole brain volume; online supplementary table S3). Direct comparison between the PCA and tAD subgroups revealed no significant between-group differences in regional grey matter correlations of olfactory performance.

## Discussion

Here we have demonstrated deficits of odour identification and categorisation in patients with PCA relative to HCs. A similar proportion (around 30%) of patients with PCA and tAD in this study had an absolute deficit of odour identification referenced to published age and gender norms and taking account of associated cognitive impairment. Olfactory impairment was similar quantitatively and qualitatively in the PCA and tAD groups. To the extent that PCA manifests underlying AD, the findings imply that olfactory impairment is a hallmark of AD pathology. It is noteworthy that only a minority of patients in both phenotypical groups here reported olfactory symptoms, suggesting that in many cases olfactory impairment is ‘subclinical’. Mean corrected odour identification scores were higher than categorisation scores in the HC and PCA groups: this unexpected finding might hold clues to the cognitive organisation of olfactory knowledge or the cognitive strategies engaged by these tests, and would warrant further study in larger populations. Odour identification tasks tend to be cognitively demanding and therefore potentially susceptible to executive and attentional deficits that accompany AD.^3^ ^13^

The deficit of odour identification identified here was associated with regional grey matter volume in a cerebral network focussed on the right anteromedial temporal lobe. The most robust neuroanatomical associations occurred in parahippocampal gyrus and entorhinal cortex: areas linked to odour identification in healthy human subjects.^6^ ^7^ Right temporal lobe degeneration has been associated previously with clinical deficits of odour recognition.^2^ Additional, less robust anatomical associations here included more posterior superior temporal and adjacent parietal areas, and premotor cortex: similar areas have been shown previously to be engaged in odour analysis,^14^ olfactory working memory^15^ and sniffing.^16^ An overlapping cerebral network has been implicated in the pathogenesis of tAD and PCA.^11^ Olfactory dysfunction may have a characteristic network signature that transcends conventional phenotypic boundaries and could potentially be used to predict AD pathology in the face of phenotypical variation. However, any claim to pathological specificity carries the important caveat that olfactory impairments have been defined for a range of non-Alzheimer neurodegenerative pathologies (notably, parkinsonian and other disorders in the frontotemporal lobar degeneration spectrum.^3–5^ ^10^ Rather than olfactory impairment per se, the signature of the impairment may be more likely to predict underlying pathology.^3^ In addition, the present study was underpowered to detect subtle behavioural or neuroanatomical differences between the syndromic groups.

This study has several limitations that suggest directions for future work. We did not assess perceptual encoding of odours: it will be important to compare associative and perceptual olfactory functions directly, to assess the extent to which these different factors contribute to olfactory impairment in AD. The patient groups studied here were relatively small: there is a need to extend the work to larger patient cohorts spanning other AD phenotypes (eg, logopenic aphasia) and in direct comparison with other neurodegenerative pathologies.^3^ Although PCA is usually attributable to AD pathology, neuropathological substrates in the present PCA cohort remain to be determined. Future longitudinal studies in different AD phenotypes (ideally, including presymptomatic carriers of AD-causing genetic mutations) will be required to assess onset and evolution of olfactory deficits.

## Supplementary Material

Web appendix

## Supplementary Material

Web appendix

## Supplementary Material

Web appendix
