# Supplementary material for: Olfactory impairment in posterior cortical atrophy
Source: J Neurol Neurosurg Psychiatry. 2013 Feb 23;84(5):588–90. doi: 10.1136/jnnp-2012-304497 (PMC3623030; doi:10.1136/jnnp-2012-304497)
Supplement: Web appendix [file jnnp-2012-304497-s1.pdf]

SUPPLEMENTARY MATERIAL

Details of behavioural assessments

Olfactory questionnaire. Prior to recruitment, all subjects completed a questionnaire (described previously)[1] detailing current olfactory symptoms and factors in their previous medical history that might impact on peripheral olfactory function (including any history of significant head injury, active disorders or surgery of upper respiratory tract, or smoking).

Olfactory assessments. Olfactory processing was assessed in all subjects using the British version of the University of Pennsylvania Smell Identification Test (UPSIT), the most widely used, quantitative assessment of olfaction.[2] The test comprises 40 odourants implanted individually on microencapsulated scratch and sniff crystals. In the standard version of the test, the subject is asked to decide on each of the 40 trials which one of four alternative written names best describes the binasally presented odour (or to guess, if no odour is perceived).

In the present study, we modified the standard UPSIT testing procedure in two ways. Firstly, on each trial prior to being presented odour names, the subject was asked to classify the source of the odour as edible or inedible (odour categorisation); the breakdown of individual UPSIT items by edibility classification (24 edible, 16 inedible) is presented in Supplementary Table S2. This odour categorisation task was motivated by evidence concerning the organisation of object processing in the visual and auditory modalities, indicating that superordinate knowledge about sensory objects can be retained even though perceptual or semantic deficits preclude explicit identification of the object. Here, we hypothesised specifically that the ability to classify odours into superordinate categories might be retained even despite degraded odour identification; and that this superordinate processing might provide an additional relevant index of central olfactory function in the target disease groups. Secondly, in order to assess odour identification, word-picture combinations were presented, and name choices were spoken by the examiner as well as presented visually: this modification was designed to reduce reliance on specific, non-olfactory (e.g., written) response cues, in order to facilitate a more accurate measure of odour processing capacity in cognitively impaired patients, as previously described.[1, 3]

Subject responses were recorded for offline analysis, and odour identification and odour categorisation performance were scored separately. No feedback was given about performance, and no time limit was imposed.

Analysis of behavioural data. Group differences in general demographic and neuropsychological characteristics were assessed using t-tests or chi-square tests. Differences between groups (PCA, tAD, HC) in olfactory performance were assessed using ANOVA. In addition to unadjusted group comparisons two adjusted analyses were conducted. The first model related raw scores on the odour identification or categorisation test to group membership (PCA, tAD, HC) with adjustment for

relevant cognitive severity measures (MMSE, executive [WASI Matrices] and verbal processing [GNT] scores), subject age and gender as covariates of no interest which could potentially influence performance on the experimental tests. The second model related odour identification scores for individual subjects after transformation to percentile scores based on published norms,[2] in order to take account of age and gender effects, to covariates of group membership and cognitive severity measures.

Raw scores on the odour identification and categorisation tasks were not directly comparable because the chance of answering correctly by guessing was higher for the categorisation test (50%) than the identification test (25%). In order to compare performance on these tasks within each group, the raw scores on each test were therefore transformed to corrected scores using a formula for scoring of multiple-choice tests[4] and differences between the test scores were then assessed using paired t-tests. The threshold for statistical significance was set at $p<0.05$. All analyses were conducted using STATA version 12.1.

Brain image acquisition and analysis

Twelve patients with PCA and eight patients with tAD had T1-weighted (MP-RAGE) volumetric MR images acquired on a 3.0T Siemens Trio scanner (Siemens) (FOV of 282 mm, 256 x 256 matrix with 208 slices; 1.1 cm isotropic resolution, with TE=2.9ms, TR=2200ms, TI=900ms) at the time of the behavioural assessments.

Voxel-based morphometry (VBM) was performed on the MR images using SPM8® (http://www.fil.ion.ucl.ac.uk/spm) following previously described procedures.[5] Briefly, native space study images were roughly aligned visually to the standard SPM8 T1 template. Then the images were segmented in to grey matter, white matter, and cerebrospinal fluid using the unified segmentation algorithm.[6] Images were then spatially normalized onto the SPM8 templates using DARTEL.[7] A study specific template was created from the MR images by creating an iteratively updated group-wise average of the grey and white matter values.[8] The grey matter and white matter segmentations were then normalised using the final transformations to the group-wise atlas and modulated to account for volume changes. The images were then smoothed with an 8 mm isotropic Gaussian kernel. Before performing statistical analysis, all images were affine-registered to Montreal Neurological Institute (MNI) stereotactic space to provide standardized coordinates for reporting of significant findings.

Linear regression was used to examine voxel-wise associations between regional grey matter volume and performance on the odour identification task across the combined patient cohort. Voxel intensity was modelled as a function of normalised odour identification scores (percentile scores), incorporating disease group (PCA and tAD), MMSE score (a measure of overall cognitive function) and total intracranial volume (calculated using a previously described procedure)[9] as covariates. A separate analysis restricted to the PCA group with the same covariates was also performed in order to assess neuroanatomical associations of odour identification performance in this target syndromic group alone. Analysis masks were created by

thresholding the group-wise average grey matter image at 0.2, so as to exclude areas with very low signal from the voxel-wise statistical analysis.

Statistical parametric maps were assessed at three voxel-wise significance thresholds: at $p<0.05$ after family-wise error (FWE) correction for multiple comparisons over the whole brain volume; at $p<0.001$ uncorrected for multiple comparisons over the whole brain volume for the purposes of characterizing the patterns observed that do not reach significance; and at $p<0.05$ after FWE correction for multiple comparisons over the anatomical small volumes of interest specified in our prior anatomical hypotheses. These anatomical small volumes were derived by manual tracing from the template brain image using MRIcron® (http://www.mccauslandcenter.sc.edu/mricro/mricron/) and comprised bilateral orbitofrontal cortices (including the orbital surface of frontal lobes and the lateral orbital gyri below the inferior frontal sulcus bilaterally), and right and left antero-medial temporal lobes anterior to Heschl's gyrus.

Supplementary results

Subject characteristics. General demographic and neuropsychological data for patients and HC subjects are summarised in Supplementary Table S1. The mean age of PCA group was significantly lower than each of the other groups; the mean age of the tAD and HC groups did not differ significantly. Subject groups did not differ significantly in gender distribution though females were relatively under-represented in the tAD group. Age and gender were included as covariates of no interest in subsequent analyses. Educational background (years of education) did not differ significantly among the groups. The patient groups did not differ in mean symptom duration or proportion of patients taking cholinesterase inhibitors at the time of testing. General neuropsychological profiles corroborated the clinical syndromic diagnosis in each of the disease groups. Both the PCA and tAD groups performed significantly worse than the HC group across cognitive domains. The PCA group performed significantly worse than the tAD group on the VOSP Objection Decision task and WASI Matrices; the tAD group performed significantly worse than the PCA group on the SRMT for words.

Olfactory symptoms. One patient in the PCA group and two patients in the tAD group reported olfactory symptoms. The PCA patient had olfactory hallucinations prior to onset of other cognitive deficits and subsequently less ability to detect odours while the two tAD patients reported loss of ability to detect odours before the onset of disease. None of the healthy control subjects reported any symptoms to suggest altered olfactory function. No subject gave a history of factors likely to have affected peripheral olfactory function.

Odour identification and categorisation. Group performance profiles on olfactory tests are summarised in Supplementary Table S1 and individual raw data are presented in Supplementary Figure S1. Although the mean identification and categorisation raw scores of PCA patients tended to be higher than those of tAD patients, there was no significant difference in scores between the syndromic groups

either before or after adjusting for age, gender and potentially relevant cognitive severity measures.

Comparing performance on odour identification and categorisation tasks within each group after correcting for guessing, mean corrected identification scores were significantly higher than mean corrected categorisation scores in the HC and PCA groups ($p<0.001$ and 0.028 respectively); no performance difference between tasks was found in the tAD group ($p=0.969$), though this is likely at least in part to have reflected the low mean scores on both tests achieved by tAD patients.

An error analysis of individual odour items in the identification test is shown in Supplementary Figure S2. The profile of odour identification errors across the set of items (expressed as the proportion of subjects making errors on each item) was qualitatively similar across the PCA, tAD and HC groups.

Neuroanatomical data. Anatomical data associated with performance on the odour identification test for the combined PCA and tAD group and for the PCA subgroup alone are summarised in Supplementary Table S3.

Supplementary references

1. Rami L, Loy CT, Hailstone J, et al. Odour identification in frontotemporal lobar degeneration. J Neurol 2007;254:431-5.
2. Doty RL, Shaman P, Dann M. Development of the University of Pennsylvania Smell Identification Test: a standardized microencapsulated test of olfactory function. Physiol Behav 1984;32:489-502.
3. Omar R, Mahoney CJ, Buckley AH, et al. Flavour identification in frontotemporal lobar degeneration. j Neurol Neurosurg Psychiatry 2012 in press.
4. Frary RB. Formula Scoring of Multiple-Choice Tests (Correction for Guessing). Educational Measurement: Issues and Practice 1988;7:33-8.
5. Rohrer JD, Ridgway GR, Modat M, et al. Distinct profiles of brain atrophy in frontotemporal lobar degeneration caused by progranulin and tau mutations. Neuroimage 2010;53:1070-6.
6. Ashburner J, Friston KJ. Unified segmentation. Neuroimage 2005;26:839-51.
7. Ashburner J. A fast diffeomorphic image registration algorithm. Neuroimage 2007;38:95-113.
8. Ashburner J, Friston KJ. Computing average shaped tissue probability templates. Neuroimage 2009;45:333-41.
9. Whitwell JL, Crum WR, Watt HC, et al. Normalization of cerebral volumes by use of intracranial volume: implications for longitudinal quantitative MR imaging. AJNR Am J Neuroradiol 2001;22:1483-9.
