# Supplementary material for: Olfactory impairment in posterior cortical atrophy
Source: J Neurol Neurosurg Psychiatry. 2013 Feb 23;84(5):588–90. doi: 10.1136/jnnp-2012-304497 (PMC3623030; doi:10.1136/jnnp-2012-304497)

Figure S1. Individual olfactory performance data

The figure shows raw scores on the odour identification test (A) and the odour categorisation test (B) of individual subjects in the healthy control (HC), posterior cortical atrophy (PCA) and typical Alzheimer's disease (tAD) groups.

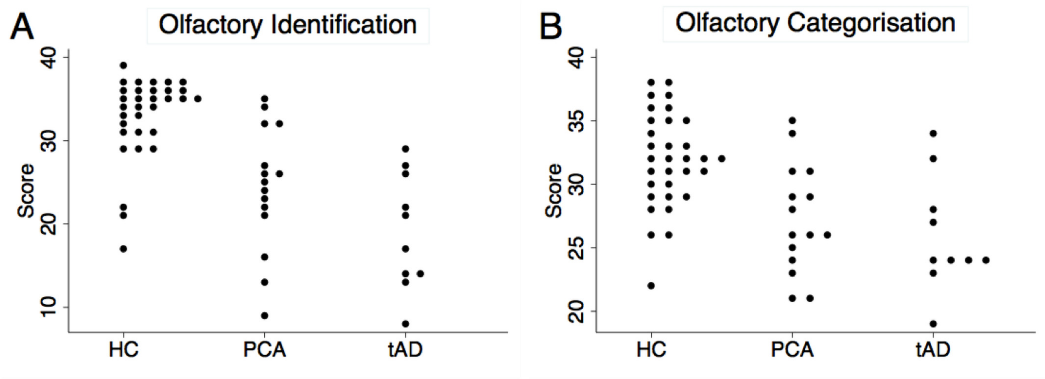

Figure S2. Error analysis profiles for individual items on the odour identification test for all groups

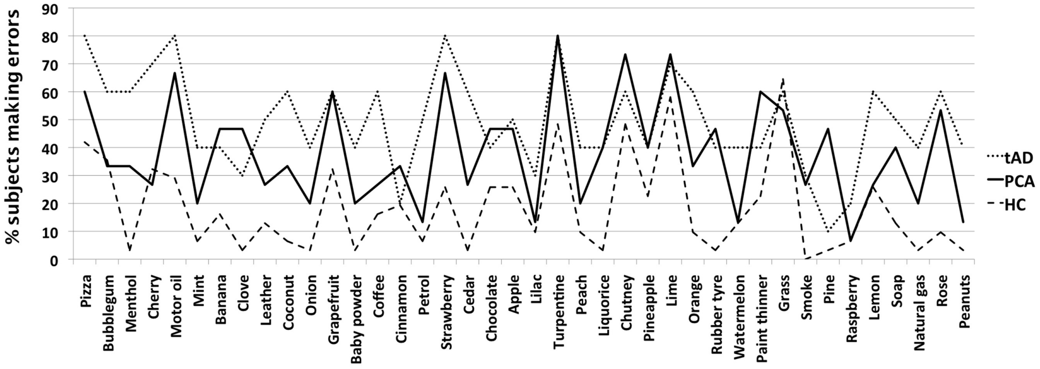

Supplement: Web appendix [file jnnp-2012-304497-s2.pdf]
