# Supplementary material for: Olfactory impairment in posterior cortical atrophy
Source: J Neurol Neurosurg Psychiatry. 2013 Feb 23;84(5):588–90. doi: 10.1136/jnnp-2012-304497 (PMC3623030; doi:10.1136/jnnp-2012-304497)
Supplement: Web appendix [file jnnp-2012-304497-s3.pdf]

SUPPLEMENTARY TABLES

Table S1. Clinical, general cognitive and olfactory function data for all groups

	**HC**<br>n = 32	**PCA**<br>n = 15	**tAD**<br>n = 10	**P value**<br>(tAD vs PCA)
**Demographic data**				
Gender (F:M)	19:13	10:5	3:7	0.082
Age (years)	68.2 (7.3)	62.6 (6.6)	69.6 (6.3)	**0.014**
Education (years)	14.2 (2.7)	12.1 (2.2)	12.6 (2.4)	0.951
Symptom duration (years)	N/A	5.84 (2.39)	7.5 (3.74)	0.187
**General cognitive functions**				
MMSE (/30)	29.88 (0.34)	19.07 (4.48)	22.9 (5.86)	0.077
SRMT (words) (/25)	24 (1.05)	20.27 (3.67)	16.8 (3.55)	**0.028**
SRMT (faces) (/25)	21.41 (2.60)	18.87 (4.53)	17.75 (3.66)	0.521
GNT (/30)	25.07 (3.45)	16.36 (6.15)	16.6 (7.99)	0.939
VOSP Object decision (/20)	18.07 (3.25)	10.6 (4.64)	16.9 (1.73)	**<0.001**
WASI Vocabulary (/80)	70.25 (5.29)	47.36 (17.04)	55.6 (16.63)	0.277
WASI matrices (/32)	24.54 (2.82)	7.64 (8.12)	19.5 (8.17)	**0.003**
**Olfactory functions**				
Identification score: raw (/40)	32.94 (4.98)	24.33 (7.51)	19.10 (6.97)	0.093
Identification score: percentile	51.81 (23.71)	14.47 (9.56)	12.3 (10.87)	0.604
Identification score: corrected* (/40)	30.58 (6.64)	19.11 (10.01)	12.13 (9.29)	0.093
Categorisation score: raw (/40)	31.91 (3.73)	27.27 (4.28)	25.9 (4.46)	0.450
Categorisation score: corrected* (/40)	23.81 (7.45)	14.53 (8.57)	11.8 (8.92)	0.450

Mean (standard deviation) values are shown. Significant differences (p<0.05) between patient groups are shown in bold. For olfactory function, p-values of unadjusted analyses are shown. Scores on all general cognitive and olfactory functions of the healthy control group were significantly different from those of each patient group. Twelve patents in the PCA group (80%) and 9 patients in the tAD group (90%) were taking a cholinesterase inhibitor at the time of testing.

Key: GNT, Graded Naming Test; HC, Healthy control; MMSE, Mini-mental state examination; n, Number; N/A, not applicable; PCA, posterior cortical atrophy; SRMT, Shorter Recognition Memory Test; tAD, typical Alzheimer's disease; VOSP, Visual Object and Space Perception Battery; WASI, Wechsler Abbreviated Scale of Intelligence. *Corrected identification and categorisation scores were transformed from the raw scores to correct for guessing.

Table S2. Edibility classification of individual UPSIT items

No	Target	Responses				Edibility
		1	2	3	4	
1	**Pizza**	Petrol	Pizza	Peanuts	Lilac	E
2	**Bubblegum**	Chutney	Bubblegum	Liniment	Watermelon	E
3	**Menthol**	Tomato	Baby powder	Strawberry	Menthol	I
4	**Cherry**	Whiskey	Honey	Lime	Cherry	E
5	**Motor oil**	Grass	Pizza	Motor oil	Pineapple	I
6	**Mint**	Dog	Mint	Peach	Cola	E
7	**Banana**	Banana	Garlic	Cherry	Motor oil	E
8	**Clove**	Baby powder	Clove	Spaghetti	Banana	E
9	**Leather**	Clove	Lilac	Leather	Apple	I
10	**Coconut**	Dog	Coconut	Cedar	Honey	E
11	**Onion**	Chocolate	Banana	Onion	Peach	E
12	**Grapefruit**	Soap	Grapefruit	Menthol	Nutmeg	E
13	**Baby powder**	Baby powder	Pineapple	Cheddar cheese	Cherry	I
14	**Coffee**	Paint thinner	Cherry	Coconut	Coffee	E
15	**Cinnamon**	Cola	Cinnamon	Pine	Coconut	E
16	**Petrol**	Rose	Lemon	Peach	Petrol	I
17	**Strawberry**	Strawberry	Chutney	Chocolate	Cedar	E
18	**Cedar**	Cedar	Petrol	Lemon	Liquorice	I
19	**Chocolate**	Lemon	Chocolate	Liquorice	Black pepper	E
20	**Apple**	Menthol	Gingerbread	Apple	Cheddar cheese	E
21	**Lilac**	Lilac	Spaghetti	Coconut	Whiskey	I
22	**Turpentine**	Turpentine	Soap	Dog	Spaghetti	I
23	**Peach**	Chocolate	Peach	Leather	Pizza	E
24	**Liquorice**	Liquorice	Watermelon	Banana	Smoke	E
25	**Chutney**	Pineapple	Chutney	Liquorice	Rose	E
26	**Pineapple**	Smoke	Whiskey	Pineapple	Onion	E
27	**Lime**	Musk	Garlic	Turpentine	Lime	E
28	**Orange**	Cheddar cheese	Orange	Bubblegum	Turpentine	E
29	**Rubber tyre**	Lime	Rubber tyre	Nutmeg	Leather	I
30	**Watermelon**	Spaghetti	Menthol	Orange	Watermelon	E
31	**Paint thinner**	Watermelon	Peanuts	Rose	Paint thinner	I
32	**Grass**	Mint	Gingerbread	Grass	Strawberry	I
33	**Smoke**	Chutney	Grass	Smoke	Peach	I
34	**Pine**	Pineapple	Smoke	Peanuts	Orange	I
35	**Raspberry**	Pizza	Turpentine	Clove	Raspberry	E
36	**Lemon**	Motor oil	Nutmeg	Rose	Lemon	E
37	**Soap**	Soap	Black pepper	Baby powder	Peanuts	I
38	**Natural gas**	Orange	Musk	Cola	Natural gas	I
39	**Rose**	Lime	Rose	Mint	Bubblegum	I
40	**Peanuts**	Peanuts	Lemon	Apple	Liquorice	E

Key: E, Edible; I, Inedible; No, Number

Table S3. Summary of anatomical regions associated with odour identification performance in the combined patient cohort and in the PCA subgroup

Anatomical region	Cluster size (voxels)	Peak MNI coordinates (mm)			T score
		x	y	z	
**Combined patient group**					
Right parahippocampal gyrus	44	21	-22	-21	5.17*
Right entorhinal cortex	129	22	8	-24	4.71*
Right temporo-parieto-occipital junction	707	58	-52	6	5.56
Right posterior cingulate cortex	156	4	-57	7	5.40
Right posterior inferior temporal gyrus	224	56	-58	-20	4.48
Right premotor cortex	41	51	3	39	4.48
Right hippocampus	71	15	-9	-17	4.30
Right posterior superior temporal sulcus	154	68	-25	-9	4.20
Left hippocampus	55	-32	-9	-20	4.18
**PCA subgroup**					
Right premotor cortex	499	39	27	45	9.78
Left premotor cortex	54	-9	23	63	7.70
Left planum temporale	112	-44	-28	4	5.66
Right temporo-parieto-occipital junction	76	60	-45	9	5.66
Right hippocampus	92	15	-6	-27	5.24

Data have been thresholded at p<0.001 uncorrected for multiple voxel-wise tests over the whole brain volume and clusters larger than 40 voxels are reported. *p value<0.05 after family-wise error (FWE) correction for small volumes of interest.
Key: mm, millimetres; MNI, Montreal Neurological Institute; PCA, posterior cortical atrophy
